# Esomeprazole attenuates inflammatory and fibrotic response in lung cells through the MAPK/Nrf2/HO1 pathway

**DOI:** 10.1186/s12950-021-00284-6

**Published:** 2021-05-19

**Authors:** Afshin Ebrahimpour, Min Wang, Li Li, Anil G. Jegga, Mark D. Bonnen, N. Tony Eissa, Ganesh Raghu, Soma Jyothula, Farrah Kheradmand, Nicola A. Hanania, Ivan O. Rosas, Yohannes T. Ghebre

**Affiliations:** 1 Department of Radiation Oncology, Baylor College of Medicine, One Baylor Plaza, Houston, TX 77030 USA; 2 Division of Biomedical Informatics, Cincinnati Children’s Hospital Medical Center, Department of Pediatrics, University of Cincinnati College of Medicine, Cincinnati, OH 45229 USA; 3 Department of Medicine, Section on Pulmonary and Critical Care Medicine, Baylor College of Medicine, Houston, TX 77030 USA; 4 Division of Pulmonary and Critical Care Medicine, Center for Interstitial Lung Disease, University of Washington, Seattle, Washington 98195 USA; 5 Department of Internal Medicine, The University of Texas Health Science Center at Houston, Houston, TX 77030 USA

**Keywords:** Esomeprazole, Proton pump inhibitors, Inflammation, Fibrosis

## Abstract

**Introduction:**

Idiopathic pulmonary fibrosis (IPF) is an orphan disease characterized by progressive loss of lung function resulting in shortness of breath and often death within 3–4 years of diagnosis. Repetitive lung injury in susceptible individuals is believed to promote chronic oxidative stress, inflammation, and uncontrolled collagen deposition. Several preclinical and retrospective clinical studies in IPF have reported beneficial outcomes associated with the use of proton pump inhibitors (PPIs) such as esomeprazole. Accordingly, we sought to investigate molecular mechanism(s) by which PPIs favorably regulate the disease process.

**Methods:**

We stimulated oxidative stress, pro-inflammatory and profibrotic phenotypes in primary human lung epithelial cells and fibroblasts upon treatment with bleomycin or transforming growth factor β (TGFβ) and assessed the effect of a prototype PPI, esomeprazole, in regulating these processes.

**Results:**

Our study shows that esomeprazole controls pro-inflammatory and profibrotic molecules through nuclear translocation of the transcription factor nuclear factor-like 2 (Nrf2) and induction of the cytoprotective molecule heme oxygenase 1 (HO1). Genetic deletion of Nrf2 or pharmacological inhibition of HO1 impaired esomeprazole-mediated regulation of proinflammatory and profibrotic molecules. Additional studies indicate that activation of Mitogen Activated Protein Kinase (MAPK) pathway is involved in the process. Our experimental data was corroborated by bioinformatics studies of an NIH chemical library which hosts gene expression profiles of IPF lung fibroblasts treated with over 20,000 compounds including esomeprazole. Intriguingly, we found 45 genes that are upregulated in IPF but downregulated by esomeprazole. Pathway analysis showed that these genes are enriched for profibrotic processes. Unbiased high throughput RNA-seq study supported antifibrotic effect of esomeprazole and revealed several novel targets.

**Conclusions:**

Taken together, PPIs may play antifibrotic role in IPF through direct regulation of the MAPK/Nrf2/HO1 pathway to favorably influence the disease process in IPF.

**Supplementary Information:**

The online version contains supplementary material available at 10.1186/s12950-021-00284-6.

## Introduction

Idiopathic pulmonary fibrosis (IPF) is a rare but deadly form of lung disease characterized by progressive loss of lung function that culminates in shortness of breath and often death within 3–4 years from the time of diagnosis. The disease has an incidence of 93.7 cases per 100,000 [1] and its prevalence increases with age with a median age at diagnosis of 60 years [1, 2]. It is believed that repetitive subclinical injuries to the lungs of susceptible individuals favor a lung microenvironment that tips the balance towards pro-oxidant, pro-inflammatory, and profibrotic processes. A few years ago, the FDA approved two drugs for the treatment of IPF; pirfenidone and nintedanib [3, 4]. Although this is a significant landmark in our quest to treat IPF, these two drugs only slow the disease progression but are unable to halt the disease or reverse established fibrosis to cure IPF. Accordingly, there is an opportunity to search for and develop more effective therapies either from libraries of new chemical entities (NCEs) or through repurposing of existing drugs approved for other indications.

Among existing drugs, proton pump inhibitors (PPIs) have promising potential to be repurposed for the treatment of IPF [5, 6]. Originally approved to reduce gastric acidity, a number of studies have linked the use of PPIs with improvement in measures of lung function leading to significantly longer transplant-free survival time in patients with well-defined IPF [7–11]. A recent official clinical practice guideline representing leading Thoracic Societies also conditionally recommended the use of PPIs for IPF [12]. However, mechanistic understanding of how PPIs regulate processes involved in lung remodeling is lacking. In this regard, we recently reported that PPIs directly inhibit an enzyme, dimethylarginine dimethylaminohydrolase (DDAH) [10], that is upregulated in lung tissues explanted from IPF patients [13, 14], and has been shown to promote experimental lung fibrosis [13].

DDAH is a cytosolic enzyme expressed in almost every mammalian cell to regulate levels of its endogenous substrate asymmetric dimethylarginine (ADMA) [15]. ADMA is a competitive inhibitor of nitric oxide (NO) synthase (NOS) including the inducible isoform (iNOS). Inducible NOS has been shown to be significantly upregulated in animal models of lung injury [16] and its genetic or pharmacological inhibition has been demonstrated to be protective in models of bleomycin-induced lung injury [10, 16, 17]. It has also been reported that IPF patients have higher levels of circulating peroxynitrite (OONO^−^); a highly reactive oxidant and nitrating molecule that is generated by iNOS during inflammatory processes [18, 19]. Overall, the DDAH/iNOS pathway is pathologically upregulated in experimental and clinical lung fibrosis, and PPIs inhibit this pathway [10].

Since esomeprazole is not a selective DDAH/iNOS inhibitor, we hypothesized that the drug may target other key biological molecules to control lung inflammation and fibrosis. Accordingly, we performed cellular and molecular studies to screen known mediators of lung inflammation and fibrosis to determine if they are targeted by the drug, and whether the drug relies on these biological targets to control inflammatory and fibrotic responses in normal and IPF-derived lung cells. To validate our cell biological studies and identify gene networks, we performed bioinformatics analysis of the NIH Library of Integrated Network-Based Cellular Signatures (LINCS) database, which hosts gene expression profiles of IPF lung fibroblasts treated with over 20,000 compounds including esomeprazole. We also performed unbiased high throughput RNA-seq studies to verify the effect of esomeprazole on inflammation- and fibrosis- related genes/pathways, and to identify novel targets.

## Materials and methods

### Cell culture

Primary human IPF lung fibroblasts were purchased from Lonza (Walkersville, MD; cat # CC-7231) and cultured in DMEM (ThermoFisher; Waltham, MA; cat # 11995065) supplemented with 10% FBS and 1% Penicillin-Streptomycin (10,000 U/ml). Human primary Bronchial/Tracheal Epithelial Cells were purchased from Lonza (cat # CC-2541) and cultured in BEGM™ Bronchial Epithelial Cell Growth Medium BulletKit™ (Lonza; cat # CC-3170). Human lung endothelial cells were also purchased from Lonza (cat # CC-2527) and cultured in EGM2-MV medium (Lonza; cat # CC-3202).

### Induction of oxidative stress and pro-inflammatory response by bleomycin

Lung epithelial cells (7 × 10^5^ cells) were seeded in 25 cm^2^ flasks and incubated in a humidified 5% CO_2_ incubator at 37 °C. Once the cells reached about 70% confluency, they were induced with bleomycin (25 μg/ml final concentration in 20 μL) or control (equal volume of water), and cultured in 4 mL fully-supplemented media in the absence or presence of different concentrations of esomeprazole (1–100 μM) for 24 h. Subsequently, the cells were harvested for gene and protein expression studies as described below.

### Induction of profibrotic response by TGFβ

Lung fibroblasts (7 × 10^5^ cells) were seeded in 25 cm^2^ flasks and incubated at 37 °C/5%CO_2_. At about 70% confluency, the conditioned media was replaced with fresh DMEM and the cells were induced with recombinant human transforming growth factor beta (TGFβ; Peprotech; Rocky Hill, NJ; cat # 100–21) for 5 days at a final concentration of 10 ng/ml in the absence or presence of various concentrations of esomeprazole (1–100 μM). The culture medium was exchanged every 48 h with fresh media containing TGFβ with or without esomeprazole. The expression of extracellular matrix (ECM) components including collagen was assessed by quantitative RT-PCR as described below.

### Effect of esomeprazole on Nrf2/HO1 antioxidant pathway

Lung epithelial cells or fibroblasts were cultured as described above. When the cells reached about 70% confluency, they were treated with various concentrations of esomeprazole (1–100 μM) for 6 to 24 h. Total RNA and protein were isolated for gene and protein expression studies, respectively. In some samples, nuclear and cytoplasmic proteins were separated for western blot studies to evaluate translocation of the transcription factor nuclear factor-like 2 (Nrf2).

### Pharmacological inhibition of HO1, Nrf2, ERK1/2, and MEK1/2

To understand the dependence of esomeprazole on heme oxygenase 1 (HO1) to control inflammatory and fibrotic processes, as well as to elucidate key signaling molecules involved in the process, we assessed HO1 and its upstream signaling that includes Nrf2, extracellular signal-regulated kinase 1/2 (ERK1/2) and MAP-ERK kinase 1/2 (MEK1/2) [20, 21] in IPF lung fibroblasts. The cells were cultured as described above and treated with pharmacological inhibitors of HO1 (Tin protoporphyrin IX; SnPPIX; Cayman Chemical; Ann Arbor, MI; cat # 16375; 1 μM final concentration), Nrf2 (Trigonelline; United States Pharmacopeia; Rockville, MD; cat # 1686411; 10 μM final concentration), or MEK1/2 (U0126; Tocris Bioscience; Bristol, UK; cat # 1144; 10 μM final concentration) for 24 h. Quantitative RT-PCR and western blot were used to assess the effect of inhibiting these targets on the upregulation of HO1 by esomeprazole. In some studies, the effect of esomeprazole on these targets including translocation of Nrf2 and phosphorylation of ERK1/2 was assessed. In other studies, the effect of HO1 inhibition (by SnPPIX) on the regulation of pro-inflammatory (TNFα, IL-1β and IL-6) and profibrotic molecules (collagen types I, III and V) by esomeprazole was assessed. To complement the pharmacological approach, lung fibroblasts were isolated from Nrf2 knockout mice (8–10 weeks old), and the dependence of esomeprazole on Nrf2 to activate HO1 was assessed. To minimize age-dependent impairment of HO1 expression in the Nrf2 knockout cells, early passage (below passage 5) fibroblasts were used.

### Gene expression analysis

Total RNA was isolated from lung fibroblasts, epithelial and endothelial cells using Direct-zol™ RNA MiniPrep Kit (Zymo Research; Irvine, CA; cat # R2050). The quality and quantity of RNA was evaluated by NanoQuant Plate™ using Tecan Spark 20 M spectrophotometer. A total of 1 μg RNA was reverse transcribed into cDNA using the SuperScript™ VILO™ Master Mix (ThermoFisher; cat # 11755) in a final reaction volume of 20 μl following the manufacturer’s protocol. The resulting cDNA was used for gene expression studies using TaqMan Gene Expression Assay (ThermoFisher; protocol # 4333458) and “best coverage” primers (ThermoFisher) on a Bio-Rad CFX96 RT-PCR system (Bio-Rad; Hercules, CA). Finally, the gene expression data was analyzed using the CFX Maestro Software (Bio-Rad) and fold changes in gene expression were calculated after normalizing to β-actin (ACTB).

### Western blot

Cytoplasmic and nuclear proteins were separated using NE-PER™ Nuclear and Cytoplasmic Extraction Kit (ThermoFisher; cat # 78833) following the manufacturer’s protocol. For the isolation of total cellular protein, cells were PBS-washed and lysed using cell lysis buffer (10 mM Na_2_HPO_4_, 0.1% Triton-X100; pH 7.4) containing 1x Halt™ Protease and Phosphatase Inhibitor Cocktail (ThermoFisher; cat # 78440). Subsequently, the cell lysate was vortexed for 1 min and incubated on ice for 30 min prior to recovering the protein by centrifugation at 16000 x g for 15 min at 4 °C. The protein concentration was determined spectrophotometrically, and equal amounts of protein (30 μg) were separated on Bis-Tris pre-cast SDS-PAGE mini-gel (ThermoFisher; cat # NP0322). The gel was transferred to polyvinylidene fluoride membrane using an iBlot™ 2 dry blotting system (ThermoFisher). Non-fat milk (5%) in Tris-buffered saline (TBS) containing 0.1% Tween-20 (TBST; pH 7.4) was used to block the membrane by incubating for 1 h at room temperature. Subsequently, the immunoblot membranes were incubated overnight with target-specific antibody including anti-HO1 (Enzo Life Sciences, Inc.; Farmingdale, NY; cat # BML-HC3001; 1:250), anti-Nrf2 (Abcam; Cambridge, MA; cat # ab62352; 1:250), anti-Keap1 (Abcam; cat # ab1194403; 1:1000), anti-phosho-ERK1/2 (Cell Signaling Technology; Danvers, MA; cat # 4370; 1:2000), and anti-phospho-MEK1/2 (Santa Cruz Biotechnology; Santa Cruz, CA; cat # sc-81,503; 1:200). For internal controls, anti-β-actin (Sigma; St. Louis, MO; cat # A2066; 1:1500) was used as control for cytoplasmic and total protein extracts, and anti-histone H3 (Abcam; cat # ab1791; 1:1000) was used as control for nuclear extracts. In some cases, total (phosphorylated plus unphosphorylated) protein was used as control to compare phosphorylation status of the same protein. Finally, the membranes were incubated for 1 h at room temperature with HRP-conjugated goat anti-rabbit (GE Healthcare; Chicago, IL; cat # NA 934 V; 1:5000), or goat anti-mouse (GE Healthcare; cat # NA 931 V; 1:5000) secondary antibody. Protein bands were visualized using Amersham ECL Prime Western Blotting Detection Reagent (Amersham Biosciences; Little Chalfont, UK; cat # RPN2236) using a ChemiDoc XRS imager system (Bio-Rad). Data was quantified using NIH’s Image J software.

### Immunofluorescence stain

Human lung epithelial cells were cultured in 6-well plates until 70% confluency and incubated further in the absence or presence of esomeprazole (1–100 μM) for 24 h. The cells were fixed with paraformaldehyde (4%) and permeabilized with 0.1% triton-X100 for 15 min prior to staining for HO1 using mouse anti-HO1 antibody (ThermoFisher; cat # MA1–112; 1:100). Secondary anti-mouse antibody (Santa Cruz; SC-362277; 1:5000) was used to detect signal. The cell membrane was stained with Alexa Fluor 488-conjugated phalloidin (ThermoFisher; cat # A12379; 1:400).

### Computational analysis

IPF gene signature containing lung transcriptomic dataset (GSE53845) [22] was queried against the LINCS database (http://www.lincscloud.org/) that contains over 1 million gene-expression profiles of cells treated with different small molecules, including esomeprazole [23]. The comparison was performed using The Connectivity Map resource [24] that was developed to connect small molecules, genes and diseases using gene-expression signatures. Z-scored differential expressions were calculated for both upregulated and downregulated gene sets, and a list of hits were ranked from highest to lowest on the basis of their match strength. Functional enrichment analysis of the inversely correlated gene sets between IPF lungs and esomeprazole-treated cells from the LINCS database was generated using ToppFun [25] and visualized using Cytoscape software [26].

### RNA-seq study

Total RNA was isolated from mouse lung fibroblasts treated with TGFβ (10 ng/mL) in the absence or presence of esomeprazole (100 μM) as described above, and integrity of the RNA was verified using Bioanalyzer 2100 (Agilent Technologies; Santa Clara, CA) at the Baylor College of Medicine, Genomic and RNA Profiling Core. Subsequently, messenger RNA (mRNA) library was prepared at Novogene Corporation (sacramento, CA) and subjected to sequencing using NovaSeq 6000 PE150 sequencing platform. Finally, the data was log-transformed, and clustered using KEGG and GO Enrichment Analyses of differentially expressed genes (DEGs). Statistical significance was set at *p* value below 0.05 (*p* < 0.05) and, fold-change threshold at 1.5.

### Statistical analysis

Data are presented as mean value ± standard error mean (SEM) from at least triplicate experiments. For the RNA-seq studies, samples were run in duplicates for comparison. Multiple groups were compared using one-way analysis of variance (ANOVA) followed by Bonferroni posttest. Comparison between two groups was performed using student’s t-test (GraphPad Prism; La Jolla, CA). Densitometric analysis of western blot data was performed using Image J and compared for statistical significance using ANOVA.

## Results

### Esomeprazole inhibits the DDAH/iNOS pathway

**Fig. 1:**
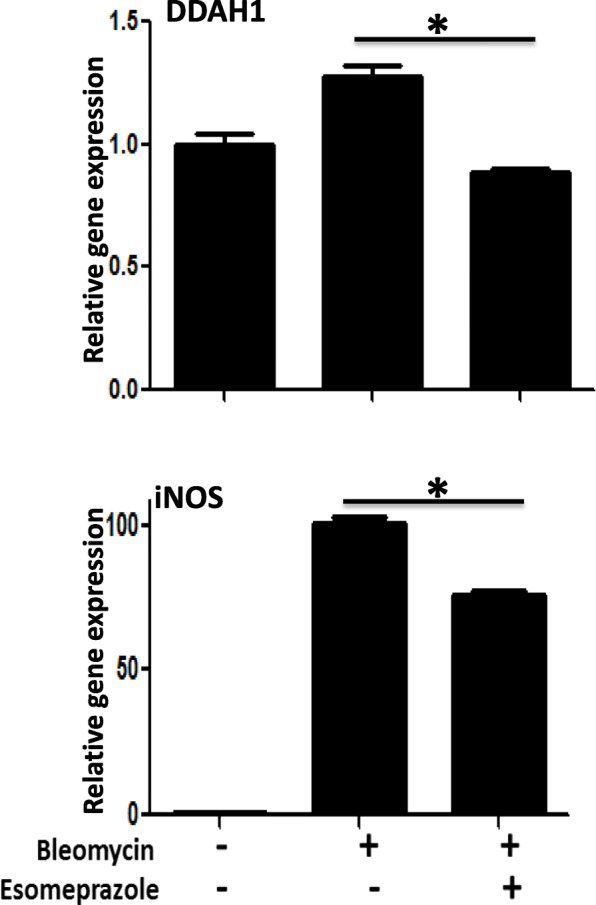
Gene expression analysis of dimethylarginine dimethylaminohydrolase (DDAH), and inducible nitric oxide synthase (iNOS) in bleomycin-exposed primary human lung epithelial cells showing downregulation of both genes by esomeprazole. Data is Mean ± SEM from triplicate experiments. *p < 0.05 in bleomycin and esomeprazole co-treatment vs. bleomycin only treated cells

The DDAH/iNOS pathway is known to be involved in aberrant lung remodeling including lung inflammation and fibrosis. Genetic or pharmacological inhibition of DDAH or iNOS has been shown to significantly attenuate fibrosis and improve lung compliance [13, 16, 17]. In this study, we found that the expression of both DDAH and iNOS were significantly downregulated by esomeprazole (Fig. 1). For example, the gene expression of iNOS was increased by about 100-fold upon induction with bleomycin, and treatment with esomeprazole decreased the induction in iNOS expression by about 25-fold (Fig. 1).

### Esomeprazole upregulates HO1 expression

**Fig. 2:**
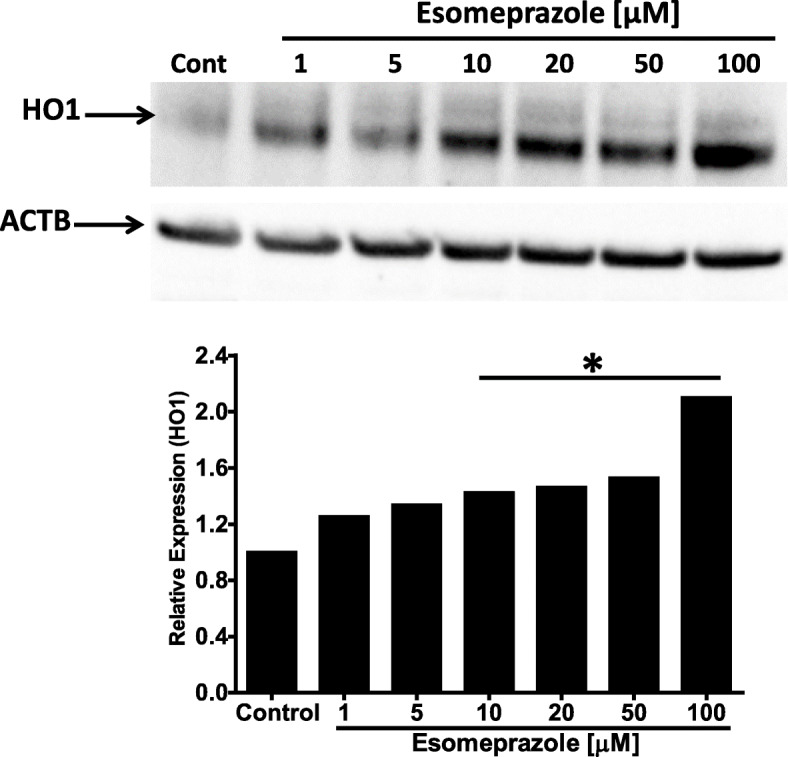
Western blot data showing increased expression of heme oxygenase 1 (HO1) protein by esomeprazole in human IPF lung fibroblasts. The cells were treated with esomeprazole (1–100 μM) for 24 h prior to isolation of total protein. Data is representative of three independent experiments. Beta actin (ACTB) is used as a loading control. Cont = control. *p < 0.05 vs control (cont)

The beneficial role of HO1 in lung homeostasis is well recognized [27–31]. As a rate-limiting enzyme in the breakdown of heme into three bioactive metabolites (i.e., carbon monoxide, bilirubin, and ferritin), HO1 is regarded as a critical mediator of lung physiology. The expression of HO1 was upregulated by esomeprazole in a dose-dependent manner (Fig. 2). As expected, the Western blot data showing induction of HO1 was reproduced by immunofluorescence (Fig. S1).

### Esomeprazole translocates Nrf2 into the nucleus

**Fig. 3:**
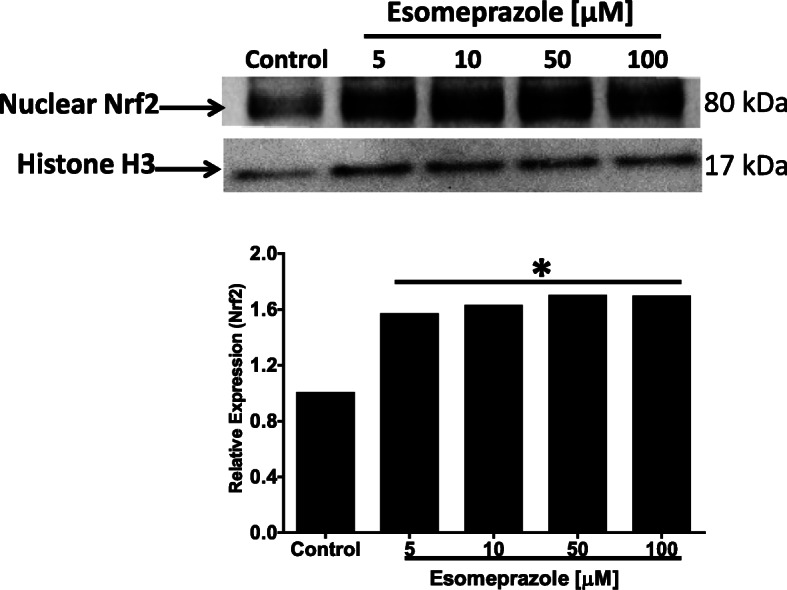
Western blot data showing nuclear translocation of nuclear factor-like 2 (Nrf2) in human IPF lung fibroblasts. The cells were treated with esomeprazole (5–100 μM) for 6 h prior to isolation of nuclear protein. Data is representative of three independent experiments. Histone H3 is used as a loading control. *p < 0.05 vs control

Physiologically, Nrf2 is kept inactive by forming a complex with Kelch ECH associating protein 1 (Keap1) in the cytoplasm of mammalian cells [32]. As a cytoprotective response to oxidative and electrophilic stress, Nrf2 is dissociated from Keap1 and translocates to the nucleus to activate antioxidant enzymes including HO1 and NADPH quinone oxidoreductase 1 (NQO1). In this study, we found that treatment of IPF lung fibroblasts with esomeprazole caused significant accumulation of Nrf2 protein in the nuclei (Fig. 3). To support this finding, we tested Nrf2 translocation in primary human lung endothelial cells and found similar results (Fig. S2). In addition to HO1, we also found that the expression of NQO1 was significantly and dose dependently upregulated by esomeprazole (Fig. S3). However, it is unlikely that the translocation of Nrf2 by esomeprazole is due to direct inhibition of Keap1 expression since we did not find changes in Keap1 protein expression (Fig. S4).

### Esomeprazole activates MAPK via phosphorylation

**Fig. 4:**
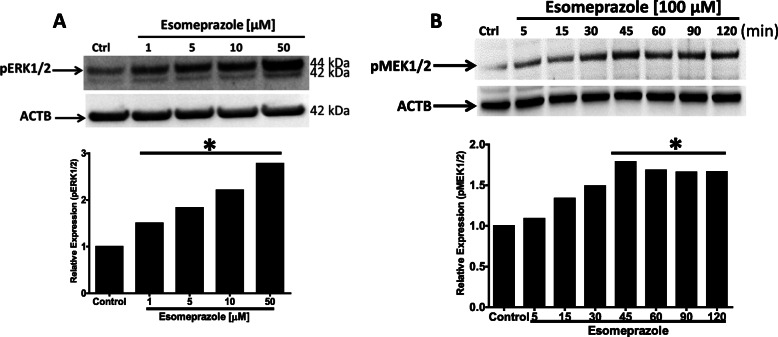
Western blot data showing phosphorylation of ERK1/2 (pERK1/2), and MEK1/2 (pMEK1/2) in human IPF lung fibroblasts treated with various concentrations of esomeprazole (1–100 μM). Data is representative of at least three independent experiments. Beta actin (ACTB) is used as loading control. Densitometric quantification of the protein bands relative to ACTB is shown in the lower panel. *p < 0.05 vs control

The mitogen-activated protein kinase (MAPK) signaling cascade includes MEK1/2 and ERK1/2; kinase enzymes which activate several other proteins via phosphorylation. For example, the ERK signaling pathway, which represents ubiquitously expressed protein kinases such as ERK1 (44 kDa) and ERK2 (42 kDa), is involved in intracellular signaling functions to influence diverse biological processes including cell adhesion, proliferation, growth, and differentiation. Phosphorylation of ERKs by MEK1/2 is necessary for activation of their kinase enzymatic activity. Activated ERK1/2 regulates phosphorylation of hundreds of regulatory molecules and transcription factors including Nrf2 [33, 34]. In our study, we found that treatment of IPF lung fibroblasts with esomeprazole phosphorylated both ERK1/2 (Fig. 4a) and MEK1/2 (Fig. 4b). This observation was also reproduced in lung epithelial (Fig. S5) and endothelial cells (Fig. S6). As expected, there was no change in the expression of total ERK protein (Fig. S7).

### Esomeprazole downregulates bleomycin-induced pro-inflammatory molecules through the HO1 pathway

**Fig. 5:**
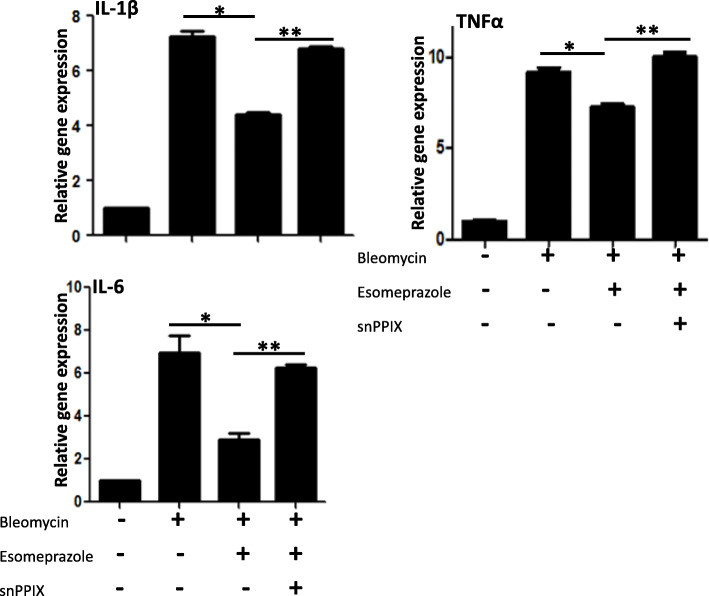
Quantitative RT-PCR (qRT-PCR) data showing upregulation of pro-inflammatory molecules TNFα, IL-1β and IL-6 in primary human lung epithelial cells treated with bleomycin for 24 h. The data also shows that treatment with esomeprazole significantly downregulated induction of the inflammatory molecules while inhibition of heme oxygenase 1 (HO1) with its selective inhibitor Tin protoporphyrin IX (SnPPIX; 1 μM) impaired the effect of esomeprazole on the expression of the inflammatory molecules. Data is Mean ± SEM from triplicate experiments. *p < 0.05 between bleomycin only vs. bleomycin + esomeprazole. **p < 0.05 between bleomycin + esomeprazole vs. bleomycin + esomeprazole + SnPPIX

The inflammatory molecule tumor necrosis factor α (TNFα) has been shown to activate latent TGFβ [35] while overexpression of interleukin 1β (IL-1β) sustains TGFβ expression and promotes lung fibrosis [36]. Here we linked the dependence of esomeprazole on HO1 to suppress TNFα and interleukins such as IL-1β and IL-6 (Fig. 5). As shown, the HO1 inhibitor SnPPIX attenuated the anti-inflammatory effects of esomeprazole.

### Esomeprazole downregulates TGFβ-induced collagen expression and the effect is blunted by pharmacological inhibition of the MAPK pathway

**Fig. 6:**
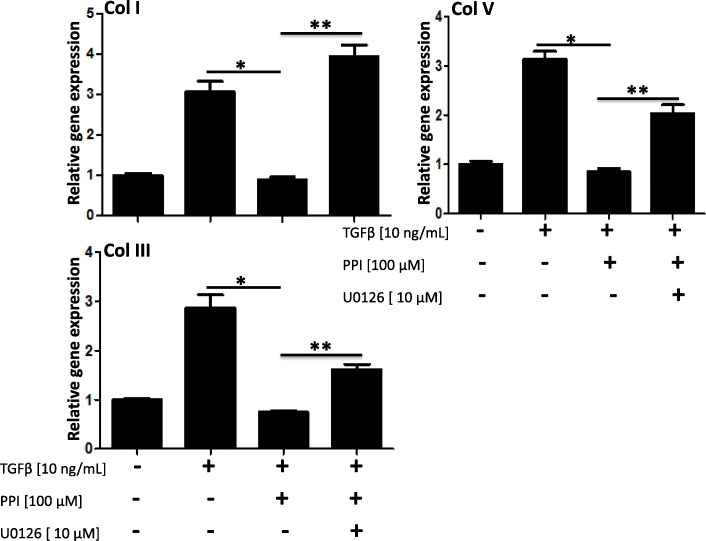
Gene expression data showing upregulation of profibrotic molecules collage I (col I), collagen III (col III), and collagen V (col V) in human IPF lung fibroblasts treated with TGFβ (10 ng/mL) for 5 days. The data also shows that treatment with the proton pump inhibitor (PPI) esomeprazole significantly downregulated induction of the fibrotic molecules while inhibition of MAP-ERK kinase 1/2 (MEK1/2) with U0126 impaired the effect of esomeprazole on all the profibrotic molecules. Data is Mean ± SEM from triplicate experiments. *p < 0.05 between TGFβ only vs. TGFβ + esomeprazole. **p < 0.05 between TGFβ + esomeprazole vs. TGFβ + esomeprazole + U0126

As major components of the ECM, several collagen types (e.g., type I, III, V) are pathologically involved in abnormal deposition of collagen and progression of lung fibrosis. Here, we report that esomeprazole significantly inhibits the expression of major collagen types and that this inhibition is mediated by the MAPK/ERK signaling (Fig. 6). As shown in the figure, pharmacological inhibition of MEK1/2 abrogated the antifibrotic effect of esomeprazole.

### Inhibition of Nrf2 impairs esomeprazole-mediated HO1 activation

**Fig. 7:**
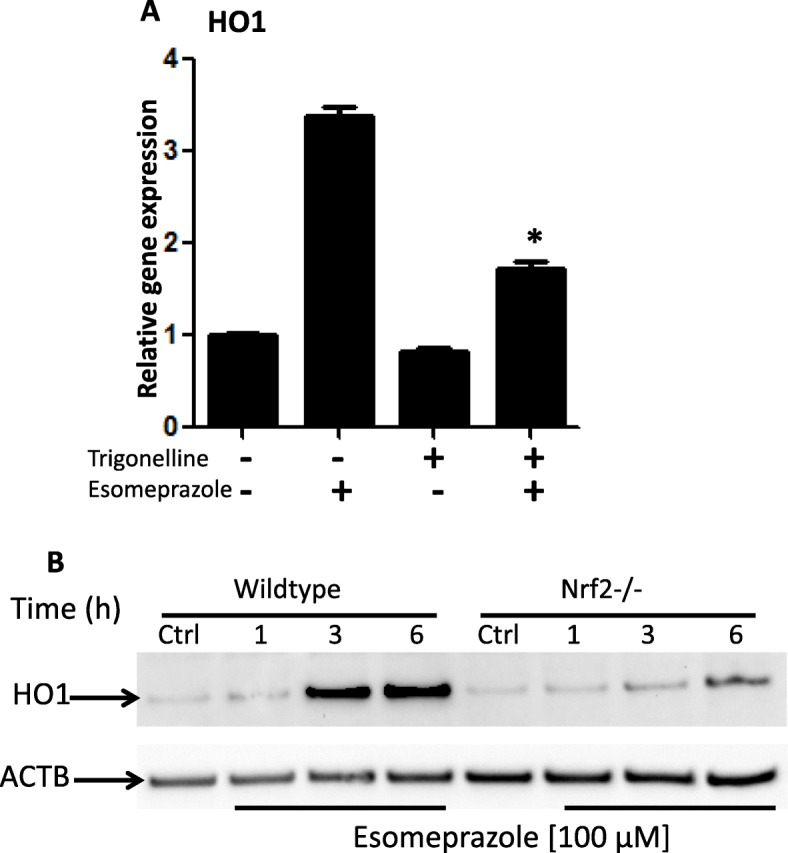
**a** Gene expression data showing upregulation of heme oxygenase 1 (HO1) by esomeprazole (100 μM) in human IPF lung fibroblasts. The data also shows that selective inhibition of nuclear factor-like 2 (Nrf2) with trigonelline (1 μM) impaired the induction of HO1 by esomeprazole. *p < 0.05 compared to esomeprazole alone. **b** Genetic deletion of Nrf2 (Nrf2−/−) impaired activation of HO1 by esomeprazole. Lung fibroblasts from Nrf2−/− mice were incubated with esomeprazole for 1–6 h. ACTB is used as a loading control. Crtl = control. Data is representative of three independent experiments

Activation of HO1 appears to be an important mechanism by which esomeprazole exerts antioxidant, anti-inflammatory and antifibrotic functions. In this study, we investigated whether activation of HO1 by esomeprazole is dependent on the transcription factor Nrf2. Pharmacological inhibition of Nrf2 with trigonelline (Fig. 7a) or genetic deletion of Nrf2 (Fig. 7b) significantly impaired the activation of HO1 by esomeprazole.

### Inhibition of MEK1/2 impairs esomeprazole-mediated HO1 activation

**Fig. 8:**
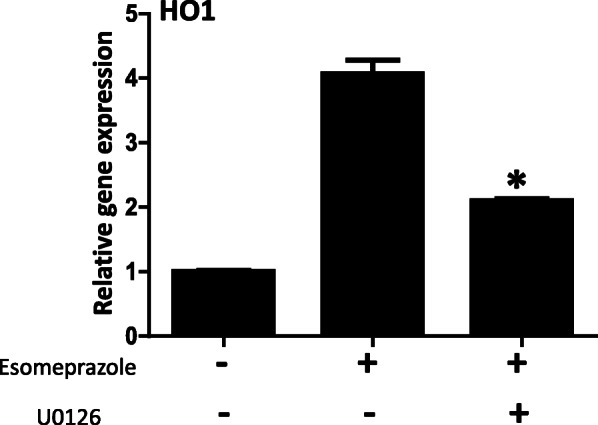
Gene expression data showing upregulation of heme oxygenase 1 (HO1) by esomeprazole (100 μM) in human IPF lung fibroblasts. The data also shows that selective inhibition of MAP-ERK kinase 1/2 (MEK1/2) with U0126 (10 μM) impaired the induction of HO1 by esomeprazole. Data is Mean ± SEM from duplicate experiments. *p < 0.05 compared to esomeprazole alone

The activation of HO1 by esomeprazole appears to be upstream of Nrf2/Keap1 towards the MAPK pathway. Accordingly, in addition to the phosphorylation of ERK1/2 by esomeprazole, pharmacological inhibition of MEK1/2 significantly impaired esomeprazole-mediated regulation of HO1 (Fig. 8).

### Esomeprazole favorably regulates several myofibroblast- and lung development- related genes

**Fig. 9:**
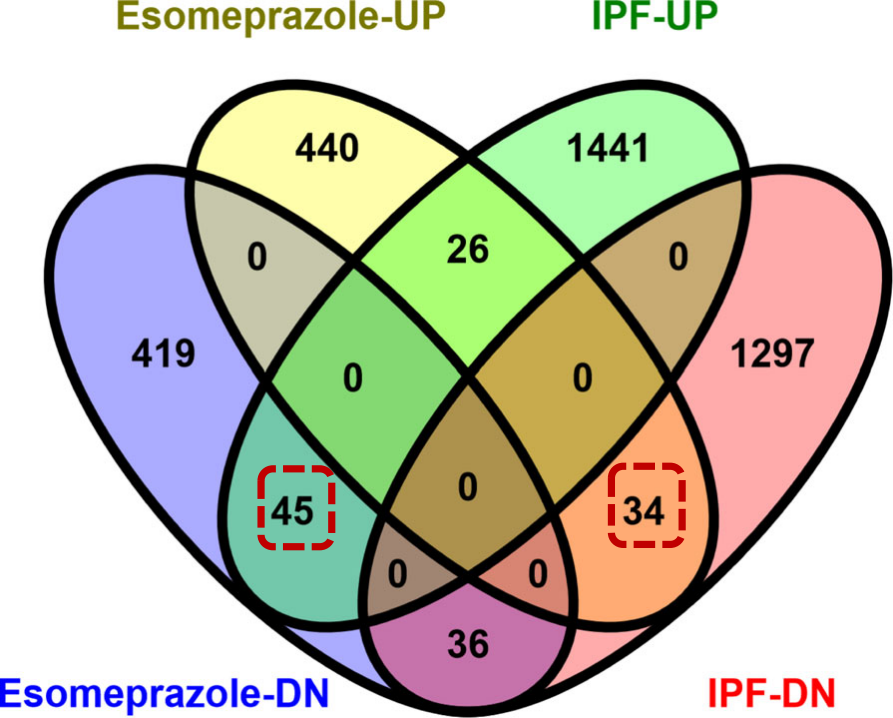
Venn diagram showing the comparison and overlap of differentially expressed genes in control and esomeprazole treated IPF lung fibroblasts from the Library of Integrated Network-Based Cellular Signatures (LINCS) database. The data shows that there are 2879 genes that are differentially expressed in the IPF cells and of which 141 genes overlap with differentially expressed genes following esomeprazole treatment. The boxed numbers indicate differentially expressed genes in IPF that are upregulated (34) or downregulated (45) following esomeprazole treatment

**Fig. 10:**
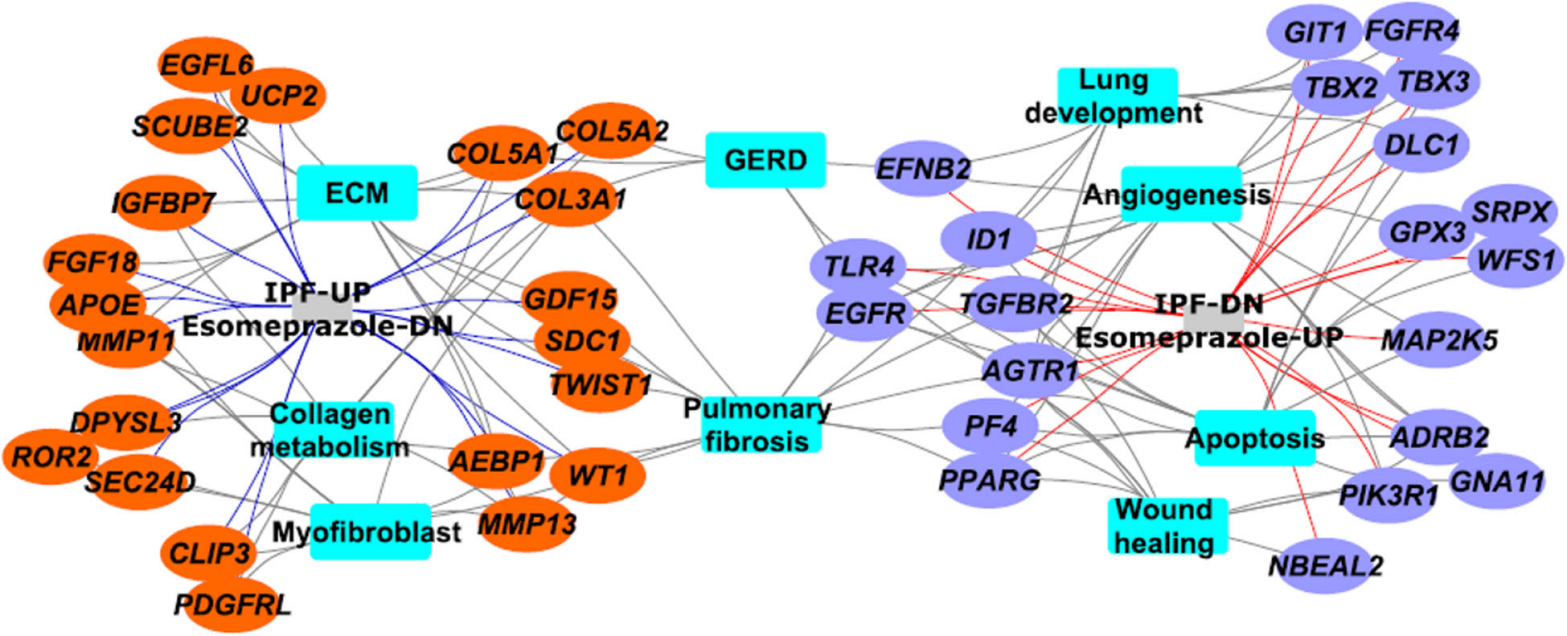
Gene networks that are activated in IPF lung fibroblasts were analyzed using ToppFun functional enrichment analysis and visualized using Cytoscape software platform. Orange colored circles represent genes that are upregulated in IPF lung fibroblasts and downregulated by esomeprazole while the purple-colored circles represent genes that are downregulated in IPF lung fibroblasts but upregulated by esomeprazole. The blue-colored squares represent enriched biological processes for the inversely correlated genes between control and esomeprazole treatment

**Fig. 11:**
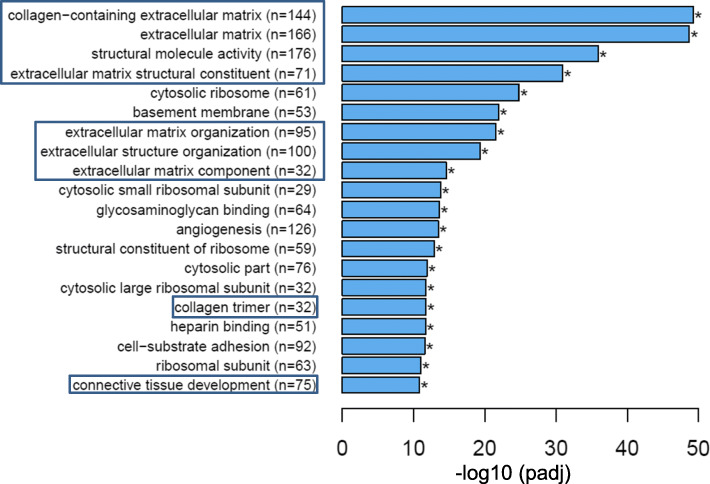
RNA-sequencing (RNA-seq) data showing significantly downregulated pathways by esomeprazole in TGFβ (10 ng/mL) stimulated mouse lung fibroblasts. The data was analyzed for statistical significance and clustered using GO Enrichment Analysis of differentially expressed genes (DEGs). Several collagen-related genes (the number of genes are shown in brackets) are downregulated by esomeprazole, and the pathways are shown in boxes. *p < 0.05 compared to TGFβ only control. Experiment was run in duplicate for each condition

Given that several retrospective clinical studies reported beneficial outcomes associated with the use of PPIs in IPF, we sought to gain mechanistic understanding of processes by which PPIs regulate lung remodeling. Accordingly, we queried the NIH’s Library of Integrated Network-Based Cellular Signatures (LINCS) [23] which hosts gene expression profiles of over 20,000 compounds including some of the most common drugs such as esomeprazole. Intriguingly, we found 45 genes that are significantly upregulated in IPF [22] but downregulated by esomeprazole (Fig. 9 and Table S1). These genes are enriched for profibrotic processes including ECM proteins such as collagen and matrix metalloproteinases (MMPs). We also found 34 lung development related genes that are downregulated in IPF but significantly upregulated by esomeprazole (Fig. 9 and Table S1). Interestingly, functional enrichment analysis using ToppFun application of the ToppGene Suite [25] showed that esomeprazole suppressed ECM, collagen metabolism and myofibroblast activation while favorably regulating genes involved in lung development, angiogenesis and wound healing (Fig. 10 and Table S1). Strikingly, RNA-seq data independently corroborated the effect of esomeprazole on ECM-related pathways including downregulation of several collagen types and other extracellular matrix components (Fig. 11, Fig. S8, and Table S2). By contrast, several lung development and wound healing related genes, including heme oxygenase 1, were upregulated by esomeprazole (Table S3).

## Discussion

### Esomeprazole is a pleiotropic molecule: activation of HO1 in lung cells

**Fig. 12:**
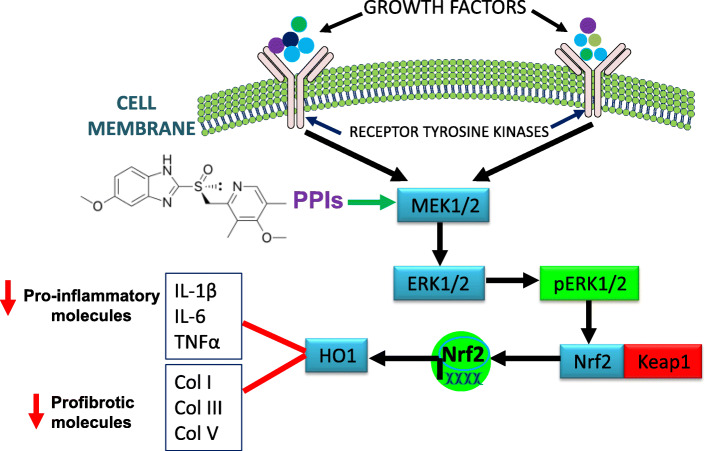
Signal transduction involving growth factors, receptor tyrosine kinases and members of the MAP kinase family. Esomeprazole-mediated activation of heme oxygenase 1 (HO1) involving phosphorylation of MEK, ERK and translocation of Nrf2 to control pro-inflammatory and profibrotic processes is illustrated

Mounting evidence indicates that PPIs possess biological activities that extend beyond suppression of gastric acidity into antioxidant, anti-inflammatory and antifibrotic properties [37–41]. The antioxidant property of PPIs is reported to be due to direct scavenging of reactive oxygen species (ROS), restoration of detoxifying enzymes such as glutathione (GSH) and HO1 [37, 39, 41–43]. As a rate-limiting enzyme in the breakdown of heme into carbon monoxide (CO), bilirubin and ferritin, HO1 plays important role in the pathobiology of lung diseases including pulmonary fibrosis. For example, CO possesses multiple biological functions including antioxidant, anti-inflammatory and bronchodilator [31, 44], and ferritin acts as an antioxidant by sequestering free iron and suppressing iron-dependent redox reaction [31]. Bilirubin is a cytoprotective molecule that has been shown to attenuate experimental lung fibrosis [45]. HO1 itself, when overexpressed, plays protective role in animal models of lung fibrosis [31, 46]. By contrast, levels of HO1 have been found to be reduced in alveolar macrophages isolated from IPF patients and in areas of active fibrosis [47, 48]. Intriguingly, our study shows that a classic PPI, esomeprazole, significantly and dose dependently upregulates the expression of HO1 (Fig. 2). The upregulation of HO1 appears to involve nuclear translocation of Nrf2 (Figs. 3 and 7) and phosphorylation of MAPK family members (Fig. 4). Among several possibilities, dissociation of Nrf2 from Keap1 may be triggered by kinases that are upstream of the transcription factor including activated (i.e. phosphorylated) ERK1/2 and MEK1/2 [33, 34] (Fig. 12). Follow up studies should interrogate signaling cascades that are upstream of ERK and MEK including Raf, Ras and tyrosine receptor kinases. Intriguingly, pharmacological inhibition or genetic deletion of Nrf2 significantly impaired HO1 activation (Fig. 7) suggesting that activation of HO1 by esomeprazole is Nrf2-dependent. Our data also shows that the anti-inflammatory action of esomeprazole is mediated by HO1 (Fig. 5), and selective inhibition of HO1 impairs esomeprazole’s effect on key inflammatory molecules. In addition, the effect of esomeprazole on the DDAH/iNOS pathway may contribute to its overall anti-inflammatory effect (Fig. 1).

### MAPK signaling pathway is involved in antifibrotic action of esomeprazole

In addition to the dependence of esomeprazole on the HO1 pathway to regulate processes involved in lung inflammation, the compound appears to depend on the MAPK signaling pathway to control TGFβ-induced expression of collagen (Fig. 6). In addition to impairing esomeprazole-mediated HO1 activation (Fig. 8), selective inhibition of MEK with U0126 appears to reduce TGFβ-induced expression of collagen compared to TGFβ only control (Fig. 6). This is consistent with an earlier report that demonstrated antifibrotic effect of MEK inhibition in experimental lung fibrosis [49]. Taken together, the activation of HO1 through phosphorylation of ERK/MEK and nuclear translocation of Nrf2, as well as inhibition of the pro-inflammatory and profibrotic DDAH/iNOS pathway contributes to the overall anti-inflammatory and antifibrotic properties of esomeprazole.

### Esomeprazole favorably regulates a network of genes involved in lung fibrosis

Consistent with our cell culture data, our bioinformatics analysis and RNA-seq studies comparing differentially expressed genes with the transcriptome of the PPI esomeprazole indicate that the antifibrotic action of esomeprazole may broadly involve suppression of ECM components including several types of collagen (e.g. collagen I, III, V) (Fig. 9 and Table S1). In fact, the RNA-seq study revealed that esomeprazole significantly downregulated over 800 extracellular matrix-related genes including collagens (Fig. 11, Fig. S8 and Table S2). The various collagen types that are upregulated in IPF fibroblasts (e.g. Figure 9) are expected to contribute to the abnormal accumulation of ECM proteins. Notably, other genes that are upregulated in IPF but found to be downregulated by esomeprazole include uncoupling protein 2 (UCP2), growth factors (e.g., FGF18), matrix metalloproteinases (e.g., MMP11, MMP13), Wilms’ Tumor 1 (WT1) and TWIST1 (Fig. 10 and Table S1). These proteins are known to play profibrotic role through increased ECM deposition, reduced collagen metabolism and/or transdifferentiation of fibroblasts into collagen-synthesizing myofibroblasts. For example, UCP2 is significantly induced by TGFβ and plays profibrotic role [50]. The role of WT1 and TWIST1 as profibrotic molecules is also well established [51–53]. By contrast, esomeprazole upregulated several lung development- and wound healing- related genes that are found to be downregulated in the control IPF lung fibroblasts (Fig. 10). For example, upregulation of Nbeal2 is expected to suppress myofibroblast infiltration and accelerate normal wound healing [54].

## Conclusions

In conclusion, PPIs in general and esomeprazole in particular may provide beneficial effect in IPF through upregulation of antioxidant, anti-inflammatory and antifibrotic molecules, as well as suppression of ECM proteins that are involved in collagen metabolism and myofibroblast activation. Given the premises of our data and the conditional recommendation of PPIs for the treatment of IPF [12], randomized controlled clinical trials evaluating the efficacy of PPIs for the treatment of IPF are warranted. However, such studies need to be designed with the intent to treat fibrosis and not gastric reflux per se. Accordingly, the dose of esomeprazole and other PPIs need to be adjusted to achieve plasma concentration of 50–100 μM in order to reliably regulate processes involved in lung remodeling. The standard antacid doses of PPIs only achieve plasma drug concentrations of 10–20 μM [55, 56]. However, higher doses of PPIs that can achieve antifibrotic concentrations can safely be administered to patients [57]. Limitations of the study include the use of bleomycin and TGFβ to induce pro-inflammatory and profibrotic processes to understand an “idiopathic” disease. Nevertheless, there is no perfect model to study IPF at preclinical level and these stimuli are often used to study the disease process in vitro and in animal models.

## Supplementary Information

**Additional file 1: Table S1.** Comparison and overlap of differentially expressed genes in control and esomeprazole treated IPF lung fibroblasts from the Library of Integrated Network-Based Cellular Signatures (LINCS) database. The data shows that there are 2879 genes that are differentially expressed in the IPF cells and of which 141 genes overlap with differentially expressed genes following esomeprazole treatment. In the first tab of the spreadsheet, the 45 esomeprazole downregulated genes are indicated in blue and the 34 esomeprazole upregulated genes are shown in orange.

**Additional file 2: Table S2.** RNA-sequencing (RNA-seq) data comparing differential gene expression in mouse lung fibroblasts stimulated with the profibrotic cytokine TGFβ (control; 10 ng/mL) or treated with TGFβ (10 ng/mL) and esomeprazole (100 μM). The fold-change, gene symbol, and description of the significantly downregulated genes (*p* < 0.05) is shown. Experiment was run in duplicate for each condition.

**Additional file 3: Table S3.** RNA-sequencing (RNA-seq) data comparing differential gene expression in mouse lung fibroblasts stimulated with the profibrotic cytokine TGFβ (control; 10 ng/mL) or treated with TGFβ (10 ng/mL) and esomeprazole (100 μM). The fold-change, gene symbol, and description of the significantly upregulated genes (*p* < 0.05) is shown. Experiment was run in duplicate for each condition.

**Additional file 4: Figure S1.** Immunofluorescence data showing increased expression of heme oxygenase 1 (HO1) protein by esomeprazole in primary human lung epithelial cells. The cells were treated with various concentrations of esomeprazole for 24 hours (1-100 μM) prior to staining with mouse anti-HO1 antibody (shown in red). The cell membrane was stained with Alexa Fluor 488-conjugated phalloidin and is shown in green. **Figure S2.** Western blot data showing nuclear translocation of nuclear factor-like 2 (Nrf2) in human lung endothelial cells. The cells were treated for 24 hours with esomeprazole (1-100 μM) or vehicle control (water) prior to isolation of nuclear protein. Data is representative of five independent experiments. Histone H3 is used as a loading control. Densitometric quantification of the protein bands relative to the housekeeping control protein histone H3 is shown in the lower panel. **Figure S3.** Quantitative RT-PCR (qRT-PCR) data showing dose-dependent upregulation of NADPH quinone oxidoreductase 1 (NQO1) by esomeprazole in human IPF lung fibroblasts. The cells were treated with various concentrations of esomeprazole for 6 hours prior to isolation of RNA for qRT-PCR. Data is Mean ± SEM from triplicate experiments. *p<0.05 compared to control. **Figure S4.** Western blot data showing no change in the protein expression of Kelch ECH associating protein 1 (Keap1) by esomeprazole in human lung epithelial cells. The cells were treated for 24 hours with various concentrations of esomeprazole (1-100 μM) or vehicle control prior to isolation of total protein. Data is representative of three independent experiments. Beta actin (ACTB) is used as a loading control. Densitometric quantification of the protein bands relative to ACTB is shown in the lower panel. **Figure S5.** Western blot data showing phosphorylation of ERK1 and ERK2 in human lung epithelial cells treated with vehicle control or various concentrations of esomeprazole (1-100 μM) for 24 hours. Data is representative of four independent experiments. Beta actin (ACTB) is used as a loading control. Densitometric quantification of the protein bands relative to ACTB is shown in the lower panel. **Figure S6.** Western blot data showing phosphorylation of ERK1 and ERK2 (pERK1/2) in human lung endothelial cells treated with vehicle control or various concentrations of esomeprazole (1-100 μM) for 24 hours. Data is representative of at least three independent experiments. Beta actin (ACTB) is used as a loading control. Densitometric quantification of the protein bands relative to ACTB is shown in the lower panel. **Figure S7.** Western blot data showing no change in the expression of total extracellular signal-regulated kinase 1/2 (ERK1/2) upon treatment of human IPF lung fibroblasts with vehicle control or esomeprazole (100 μM) for up to 2 hours. Data is representative of three independent experiments. Beta actin (ACTB) is used as a loading control. Densitometric quantification of the protein bands relative to ACTB is shown in the lower panel. **Figure S8.** Volcano plot of RNA-seq data from mouse lung fibroblasts stimulated with the profibrotic cytokine TGFβ (control; 10 ng/mL) or treated with TGFβ (10 ng/mL) and esomeprazole (100 μM). The plot shows the total number of significantly upregulated (1876; red) and downregulated (2035; green) genes by esomeprazole. The total number of unchanged genes (17,114) is shown in blue.

## Data Availability

The datasets and materials used and analyzed during the current study are available from the corresponding author on reasonable request.

## References

[1] Raghu G, Chen SY, Yeh WS, Maroni B, Li Q, Lee YC, Collard HR (2014). Idiopathic pulmonary fibrosis in US Medicare beneficiaries aged 65 years and older: incidence, prevalence, and survival, 2001-11. Lancet Respir Med.

[2] Hodgson U, Laitinen T, Tukiainen P (2002). Nationwide prevalence of sporadic and familial idiopathic pulmonary fibrosis: evidence of founder effect among multiplex families in Finland. Thorax.

[3] King TE, Bradford WZ, Castro-Bernardini S, Fagan EA, Glaspole I, Glassberg MK, Gorina E, Hopkins PM, Kardatzke D, Lancaster L, Lederer DJ, Nathan SD, Pereira CA, Sahn SA, Sussman R, Swigris JJ, Noble PW (2014). A phase 3 trial of pirfenidone in patients with idiopathic pulmonary fibrosis. N Engl J Med.

[4] Richeldi L, du Bois RM, Raghu G, Azuma A, Brown KK, Costabel U, Cottin V, Flaherty KR, Hansell DM, Inoue Y, Kim DS, Kolb M, Nicholson AG, Noble PW, Selman M, Taniguchi H, Brun M, le Maulf F, Girard M, Stowasser S, Schlenker-Herceg R, Disse B, Collard HR (2014). Efficacy and safety of nintedanib in idiopathic pulmonary fibrosis. N Engl J Med.

[5] Ghebre YT (2018). Proton pump inhibitors in IPF: a call for clinical trials. Front Pharmacol.

[6] Ghebre YT, Raghu G (2016). Idiopathic pulmonary fibrosis: novel concepts of proton pump inhibitors as Antifibrotic drugs. Am J Respir Crit Care Med.

[7] Raghu G, Freudenberger TD, Yang S (2006). High prevalence of abnormal acid gastro-oesophageal reflux in idiopathic pulmonary fibrosis. Eur Respir J.

[8] Lee JS, Ryu JH, Elicker BM, Lydell CP, Jones KD, Wolters PJ, King TE, Collard HR (2011). Gastroesophageal reflux therapy is associated with longer survival in patients with idiopathic pulmonary fibrosis. Am J Respir Crit Care Med.

[9] Lee J, Collard HR, Anstrom KJ, Martinez FJ, Noth I, Roberts RS, Yow E, Raghu G, IPFnet Investigators (2013). Anti-acid treatment and disease progression in idiopathic pulmonary fibrosis: an analysis of data from three randomised controlled trials. Lancet Respir Med.

[10] Ghebremariam YT, Cooke JP, Gerhart W, Griego C, Brower JB, Doyle-Eisele M, Moeller BC, Zhou Q, Ho L, de Andrade J, Raghu G, Peterson L, Rivera A, Rosen GD (2015). Pleiotropic effect of the proton pump inhibitor esomeprazole leading to suppression of lung inflammation and fibrosis. J Transl Med.

[11] Lee CM, Lee DH, Ahn BK, Hwang JJ, Yoon H, Shin CM, Park YS, Kim N (2016). Protective effect of proton pump inhibitor for survival in patients with Gastroesophageal reflux disease and idiopathic pulmonary fibrosis. J Neurogastroenterol Motil.

[12] Raghu G, Rochwerg B, Zhang Y, Garcia CA, Azuma A, Behr J, Brozek JL, Collard HR, Cunningham W, Homma S, Johkoh T, Martinez FJ, Myers J, Protzko SL, Richeldi L, Rind D, Selman M, Theodore A, Wells AU, Hoogsteden H, Schünemann HJ, American Thoracic Society, European Respiratory society, Japanese Respiratory Society, Latin American Thoracic Association (2015). An official ATS/ERS/JRS/ALAT clinical practice guideline: treatment of idiopathic pulmonary fibrosis. An update of the 2011 clinical practice guideline. Am J Respir Crit Care Med.

[13] Pullamsetti SS, Savai R, Dumitrascu R (2011). The role of dimethylarginine dimethylaminohydrolase in idiopathic pulmonary fibrosis. Sci Transl Med.

[14] Janssen W, Pullamsetti SS, Cooke J, Weissmann N, Guenther A, Schermuly RT (2013). The role of dimethylarginine dimethylaminohydrolase (DDAH) in pulmonary fibrosis. J Pathol.

[15] Palm F, Onozato ML, Luo Z, Wilcox CS (2007). Dimethylarginine dimethylaminohydrolase (DDAH): expression, regulation, and function in the cardiovascular and renal systems. Am J Physiol Heart Circ Physiol.

[16] Seimetz M, Parajuli N, Pichl A, Veit F, Kwapiszewska G, Weisel FC, Milger K, Egemnazarov B, Turowska A, Fuchs B, Nikam S, Roth M, Sydykov A, Medebach T, Klepetko W, Jaksch P, Dumitrascu R, Garn H, Voswinckel R, Kostin S, Seeger W, Schermuly RT, Grimminger F, Ghofrani HA, Weissmann N (2011). Inducible NOS inhibition reverses tobacco-smoke-induced emphysema and pulmonary hypertension in mice. Cell.

[17] Genovese T, Cuzzocrea S, Di Paola R (2005). Inhibition or knock out of inducible nitric oxide synthase result in resistance to bleomycin-induced lung injury. Respir Res.

[18] Saleh D, Barnes PJ, Giaid A (1997). Increased production of the potent oxidant peroxynitrite in the lungs of patients with idiopathic pulmonary fibrosis. Am J Respir Crit Care Med.

[19] Montaldo C, Cannas E, Ledda M (2002). Bronchoalveolar glutathione and nitrite/nitrate in idiopathic pulmonary fibrosis and sarcoidosis. Sarcoidosis Vasc Diffuse Lung Dis.

[20] Zipper LM, Mulcahy RT (2003). Erk activation is required for Nrf2 nuclear localization during pyrrolidine dithiocarbamate induction of glutamate cysteine ligase modulatory gene expression in HepG2 cells. Toxicol Sci.

[21] Naidu S, Vijayan V, Santoso S, Kietzmann T, Immenschuh S (2009). Inhibition and genetic deficiency of p38 MAPK up-regulates heme oxygenase-1 gene expression via Nrf2. J Immunol.

[22] DePianto DJ, Chandriani S, Abbas AR (2015). Heterogeneous gene expression signatures correspond to distinct lung pathologies and biomarkers of disease severity in idiopathic pulmonary fibrosis. Thorax.

[23] Subramanian A, Narayan R, Corsello SM, Peck DD, Natoli TE, Lu X, Gould J, Davis JF, Tubelli AA, Asiedu JK, Lahr DL, Hirschman JE, Liu Z, Donahue M, Julian B, Khan M, Wadden D, Smith IC, Lam D, Liberzon A, Toder C, Bagul M, Orzechowski M, Enache OM, Piccioni F, Johnson SA, Lyons NJ, Berger AH, Shamji AF, Brooks AN, Vrcic A, Flynn C, Rosains J, Takeda DY, Hu R, Davison D, Lamb J, Ardlie K, Hogstrom L, Greenside P, Gray NS, Clemons PA, Silver S, Wu X, Zhao WN, Read-Button W, Wu X, Haggarty SJ, Ronco LV, Boehm JS, Schreiber SL, Doench JG, Bittker JA, Root DE, Wong B, Golub TR (2017). A next generation connectivity map: L1000 platform and the first 1,000,000 profiles. Cell.

[24] Lamb J, Crawford ED, Peck D, Modell JW, Blat IC, Wrobel MJ, Lerner J, Brunet JP, Subramanian A, Ross KN, Reich M, Hieronymus H, Wei G, Armstrong SA, Haggarty SJ, Clemons PA, Wei R, Carr SA, Lander ES, Golub TR (2006). The connectivity map: using gene-expression signatures to connect small molecules, genes, and disease. Science.

[25] Chen J, Bardes EE, Aronow BJ, Jegga AG (2009). ToppGene suite for gene list enrichment analysis and candidate gene prioritization. Nucleic Acids Res.

[26] Shannon P, Markiel A, Ozier O, Baliga NS, Wang JT, Ramage D, Amin N, Schwikowski B, Ideker T (2003). Cytoscape: a software environment for integrated models of biomolecular interaction networks. Genome Res.

[27] Morse D, Choi AM (2002). Heme oxygenase-1: the “emerging molecule” has arrived. Am J Respir Cell Mol Biol.

[28] Morse D, Choi AM (2005). Heme oxygenase-1: from bench to bedside. Am J Respir Crit Care Med.

[29] Slebos DJ, Ryter SW, Choi AM (2003). Heme oxygenase-1 and carbon monoxide in pulmonary medicine. Respir Res.

[30] Morse D, Lin L, Choi AM (2009). Heme oxygenase-1, a critical arbitrator of cell death pathways in lung injury and disease. Free Radic Biol Med.

[31] Constantin M, Choi AJ, Cloonan SM (2012). Therapeutic potential of heme oxygenase-1/carbon monoxide in lung disease. Int J Hypertens.

[32] Kansanen E, Kuosmanen SM, Leinonen H, Levonen AL (2013). The Keap1-Nrf2 pathway: mechanisms of activation and dysregulation in cancer. Redox Biol.

[33] Busca R, Pouyssegur J, Lenormand P (2016). ERK1 and ERK2 map kinases: specific roles or functional redundancy?. Front Cell Dev Biol.

[34] Cullinan SB, Zhang D, Hannink M, Arvisais E, Kaufman RJ, Diehl JA (2003). Nrf2 is a direct PERK substrate and effector of PERK-dependent cell survival. Mol Cell Biol.

[35] Sullivan DE, Ferris M, Pociask D, Brody AR (2008). The latent form of TGFbeta (1) is induced by TNFalpha through an ERK specific pathway and is activated by asbestos-derived reactive oxygen species in vitro and in vivo. J Immunotoxicol.

[36] Kolb M, Margetts PJ, Anthony DC, Pitossi F, Gauldie J (2001). Transient expression of IL-1beta induces acute lung injury and chronic repair leading to pulmonary fibrosis. J Clin Invest.

[37] Namazi MR, Jowkar F (2008). A succinct review of the general and immunological pharmacologic effects of proton pump inhibitors. J Clin Pharm Ther.

[38] Kedika RR, Souza RF, Spechler SJ (2009). Potential anti-inflammatory effects of proton pump inhibitors: a review and discussion of the clinical implications. Dig Dis Sci.

[39] Becker JC, Grosser N, Waltke C, Schulz S, Erdmann K, Domschke W, Schröder H, Pohle T (2006). Beyond gastric acid reduction: proton pump inhibitors induce heme oxygenase-1 in gastric and endothelial cells. Biochem Biophys Res Commun.

[40] Sasaki T, Nakayama K, Yasuda H, Yamaya M (2011). A new strategy with proton pump inhibitors for the prevention of acute exacerbations in COPD. Ther Adv Respir Dis.

[41] Biswas K, Bandyopadhyay U, Chattopadhyay I, Varadaraj A, Ali E, Banerjee RK (2003). A novel antioxidant and antiapoptotic role of omeprazole to block gastric ulcer through scavenging of hydroxyl radical. J Biol Chem.

[42] Lapenna D, de Gioia S, Ciofani G (1996). Antioxidant properties of omeprazole. FEBS Lett.

[43] Simon WA, Sturm E, Hartmann HJ, Weser U (2006). Hydroxyl radical scavenging reactivity of proton pump inhibitors. Biochem Pharmacol.

[44] Otterbein LE, Bach FH, Alam J, Soares M, Tao Lu H, Wysk M, Davis RJ, Flavell RA, Choi AMK (2000). Carbon monoxide has anti-inflammatory effects involving the mitogen-activated protein kinase pathway. Nat Med.

[45] Wang HD, Yamaya M, Okinaga S (2002). Bilirubin ameliorates bleomycin-induced pulmonary fibrosis in rats. Am J Respir Crit Care Med.

[46] Tsuburai T, Suzuki M, Nagashima Y, Suzuki S, Inoue S, Hashiba T, Ueda A, Ikehara K, Matsuse T, Ishigatsubo Y (2002). Adenovirus-mediated transfer and overexpression of heme oxygenase 1 cDNA in lung prevents bleomycin-induced pulmonary fibrosis via a Fas-Fas ligand-independent pathway. Hum Gene Ther.

[47] Lakari E, Pylkas P, Pietarinen-Runtti P (2001). Expression and regulation of hemeoxygenase 1 in healthy human lung and interstitial lung disorders. Hum Pathol.

[48] Ye Q, Dalavanga Y, Poulakis N, Sixt SU, Guzman J, Costabel U (2008). Decreased expression of haem oxygenase-1 by alveolar macrophages in idiopathic pulmonary fibrosis. Eur Respir J.

[49] Madala SK, Schmidt S, Davidson C, Ikegami M, Wert S, Hardie WD (2012). MEK-ERK pathway modulation ameliorates pulmonary fibrosis associated with epidermal growth factor receptor activation. Am J Respir Cell Mol Biol.

[50] Jiang L, Qiu W, Zhou Y, Wen P, Fang L, Cao H, Zen K, He W, Zhang C, Dai C, Yang J (2013). A microRNA-30e/mitochondrial uncoupling protein 2 axis mediates TGF-beta1-induced tubular epithelial cell extracellular matrix production and kidney fibrosis. Kidney Int.

[51] Sontake V, Shanmukhappa SK, DiPasquale BA (2015). Fibrocytes regulate Wilms tumor 1-positive cell accumulation in severe fibrotic lung disease. J Immunol.

[52] Sontake V, Kasam RK, Sinner D, Korfhagen TR, Reddy GB, White ES, et al. Wilms' tumor 1 drives fibroproliferation and myofibroblast transformation in severe fibrotic lung disease. JCI Insight. 2018;3(16). 10.1172/jci.insight.121252. 10.1172/jci.insight.121252 PMC6141179 30135315

[53] Mammoto T, Jiang A, Jiang E, Mammoto A (2016). Role of Twist1 phosphorylation in angiogenesis and pulmonary fibrosis. Am J Respir Cell Mol Biol.

[54] Deppermann C, Cherpokova D, Nurden P, Schulz JN, Thielmann I, Kraft P, Vögtle T, Kleinschnitz C, Dütting S, Krohne G, Eming SA, Nurden AT, Eckes B, Stoll G, Stegner D, Nieswandt B (2013). Gray platelet syndrome and defective thrombo-inflammation in Nbeal2-deficient mice. J Clin Invest.

[55] Shin JM, Sachs G (2008). Pharmacology of proton pump inhibitors. Curr Gastroenterol Rep.

[56] Ghebremariam YT, LePendu P, Lee JC, Erlanson DA, Slaviero A, Shah NH, Leiper J, Cooke JP (2013). Unexpected effect of proton pump inhibitors: elevation of the cardiovascular risk factor asymmetric dimethylarginine. Circulation.

[57] Brana I, Ocana A, Chen EX, Razak ARA, Haines C, Lee C, Douglas S, Wang L, Siu LL, Tannock IF, Bedard PL (2014). A phase I trial of pantoprazole in combination with doxorubicin in patients with advanced solid tumors: evaluation of pharmacokinetics of both drugs and tissue penetration of doxorubicin. Investig New Drugs.

