# Supplementary figures and images for: Esomeprazole attenuates inflammatory and fibrotic response in lung cells through the MAPK/Nrf2/HO1 pathway
Source: J Inflamm (Lond). 2021 May 19;18:17. doi: 10.1186/s12950-021-00284-6 (PMC8136131; doi:10.1186/s12950-021-00284-6)

## Supplemental Data

Figure S1

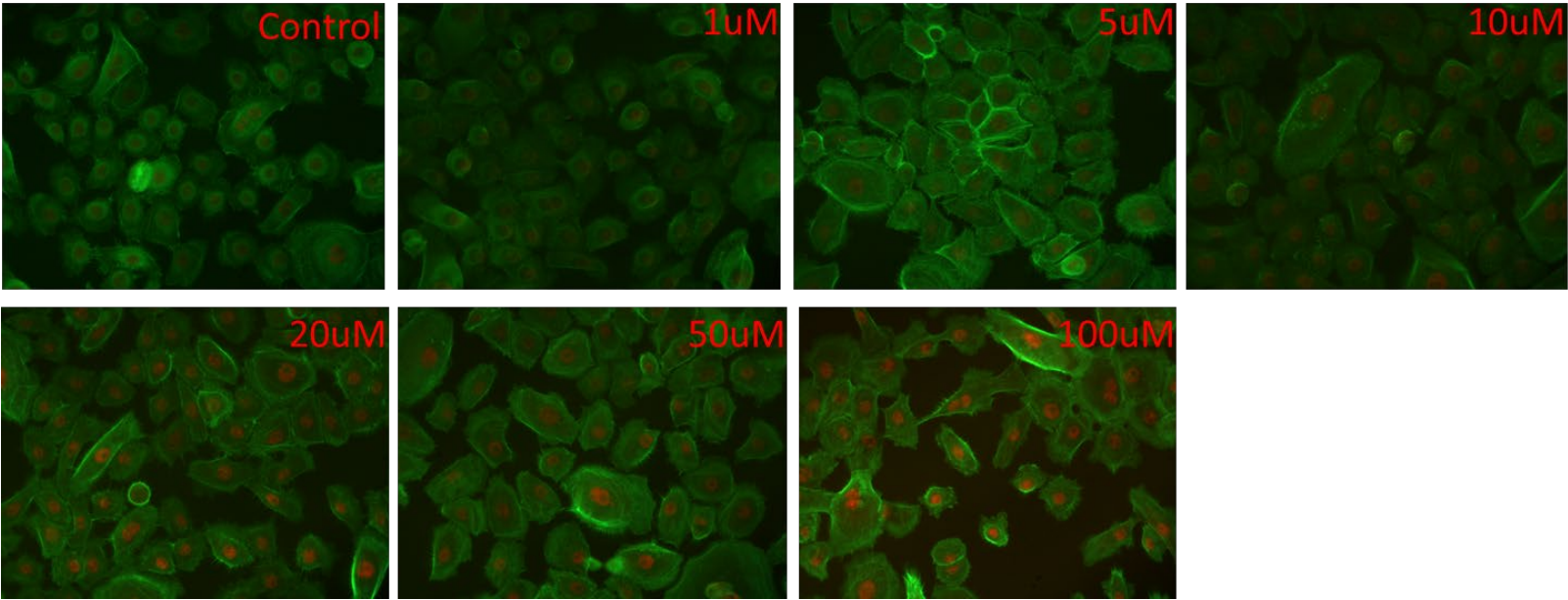

Figure S2

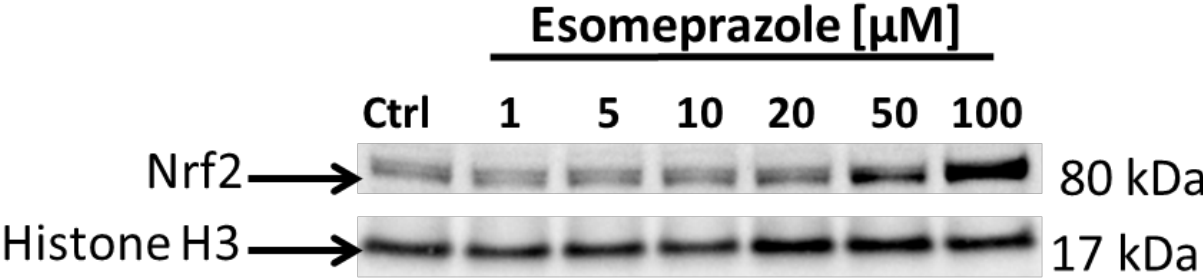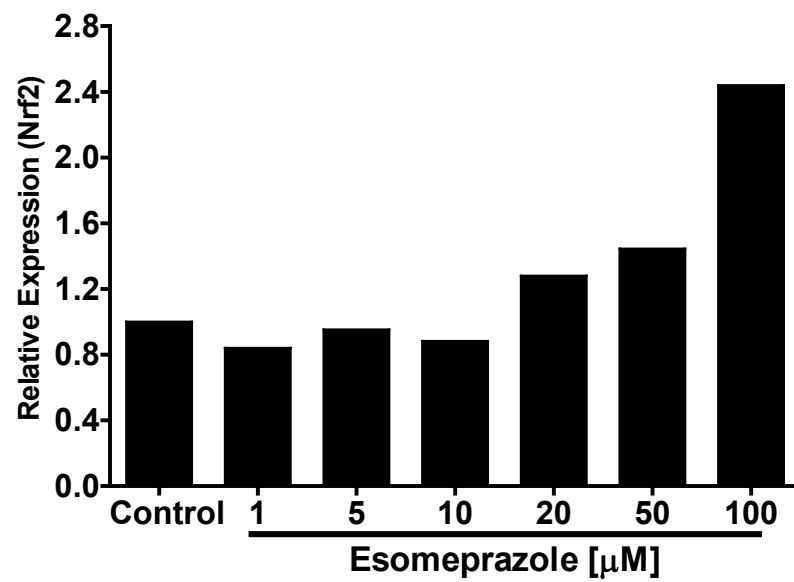

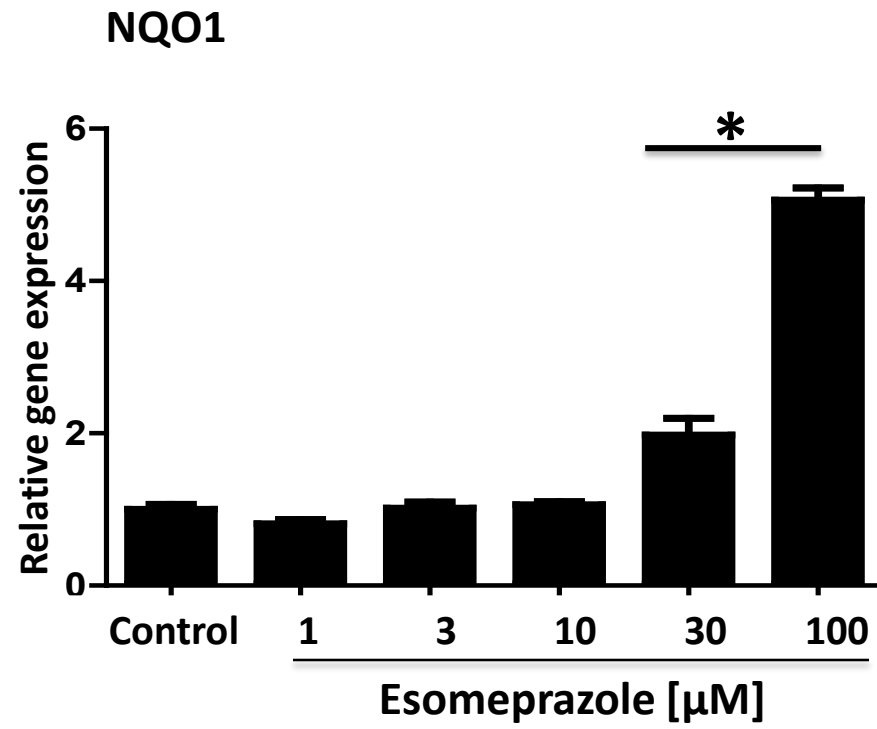

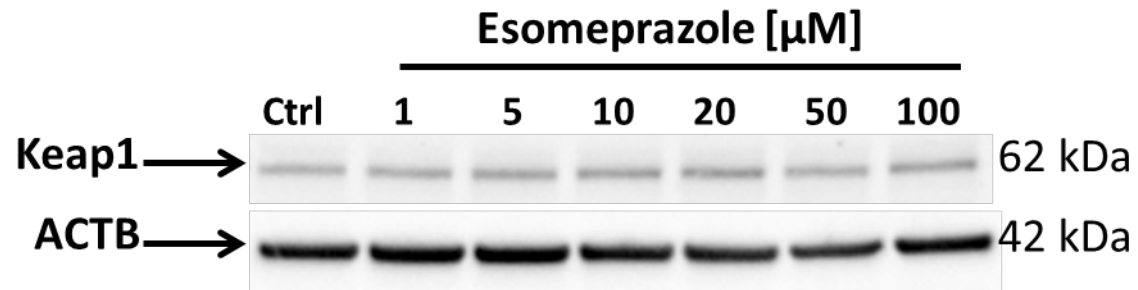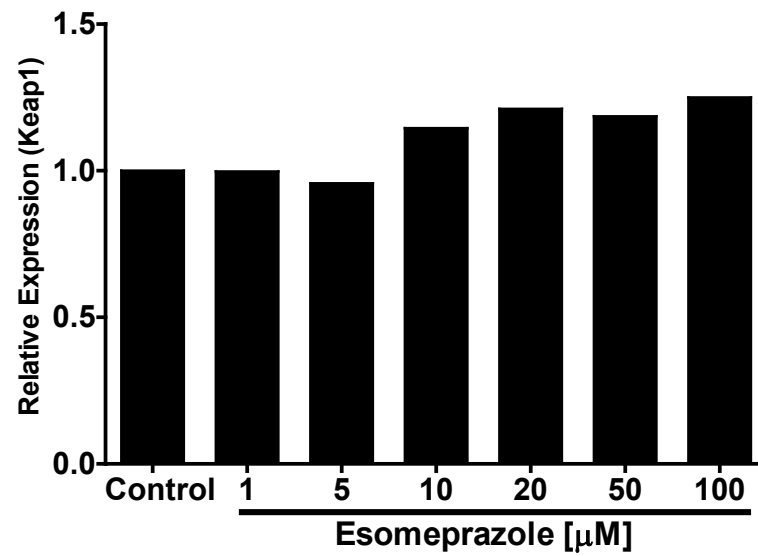

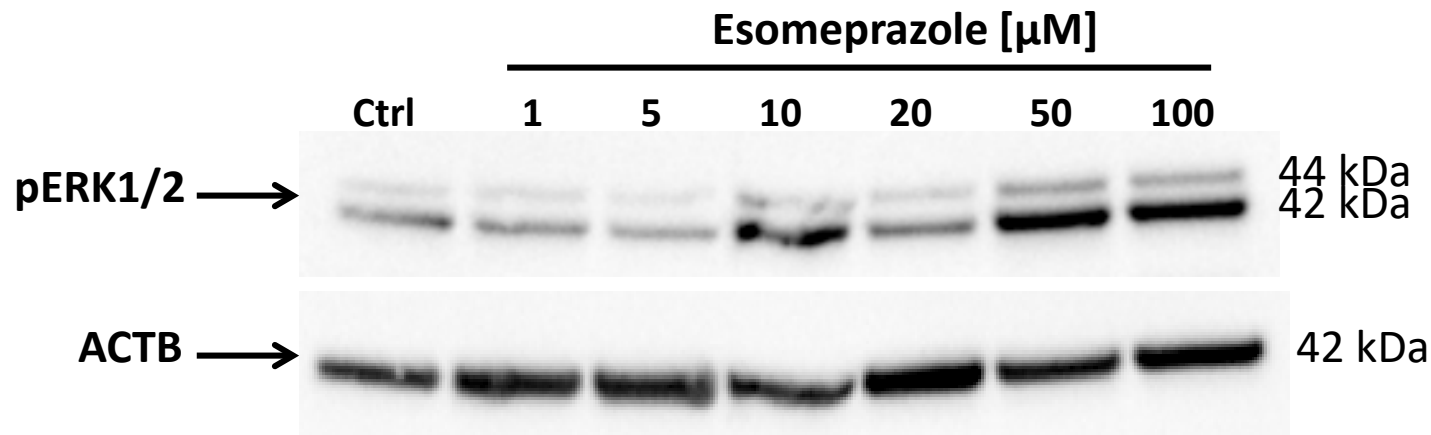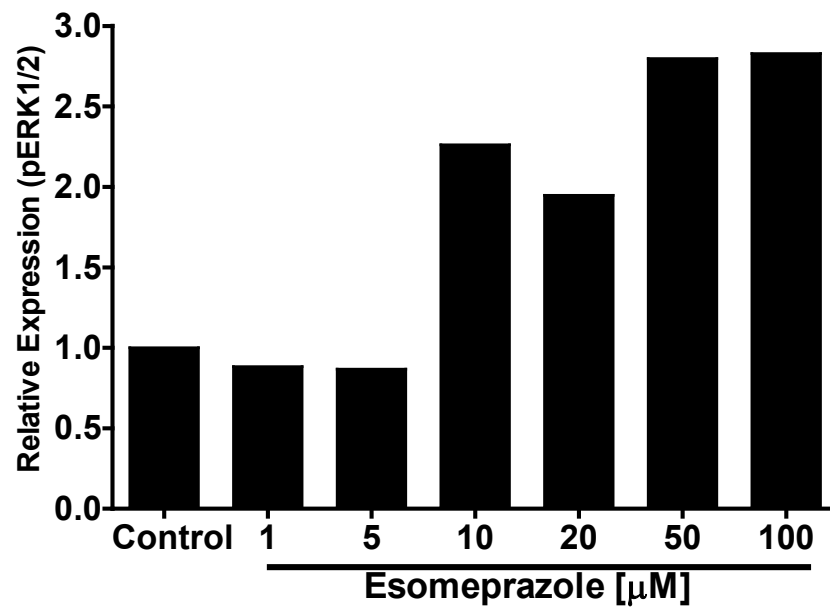

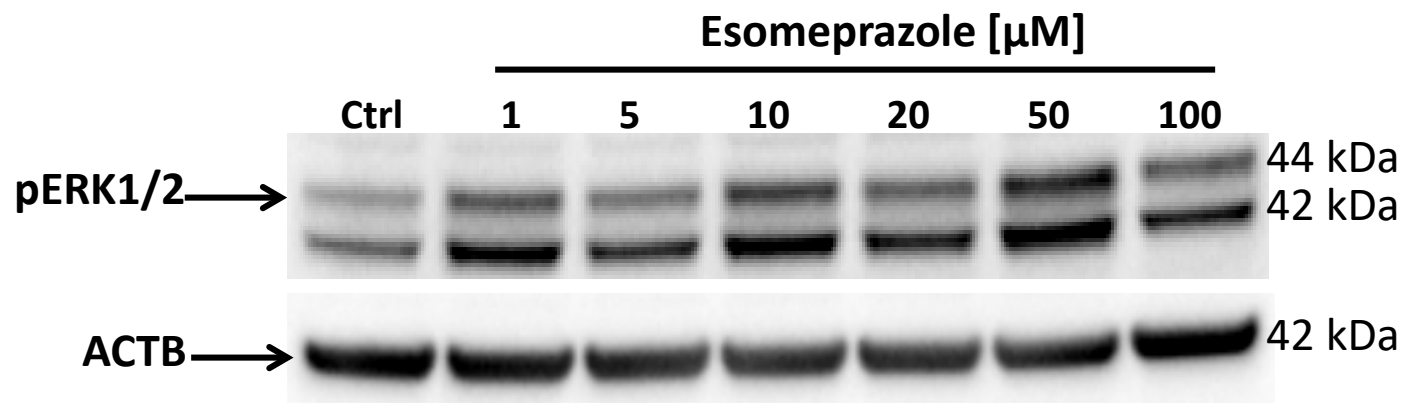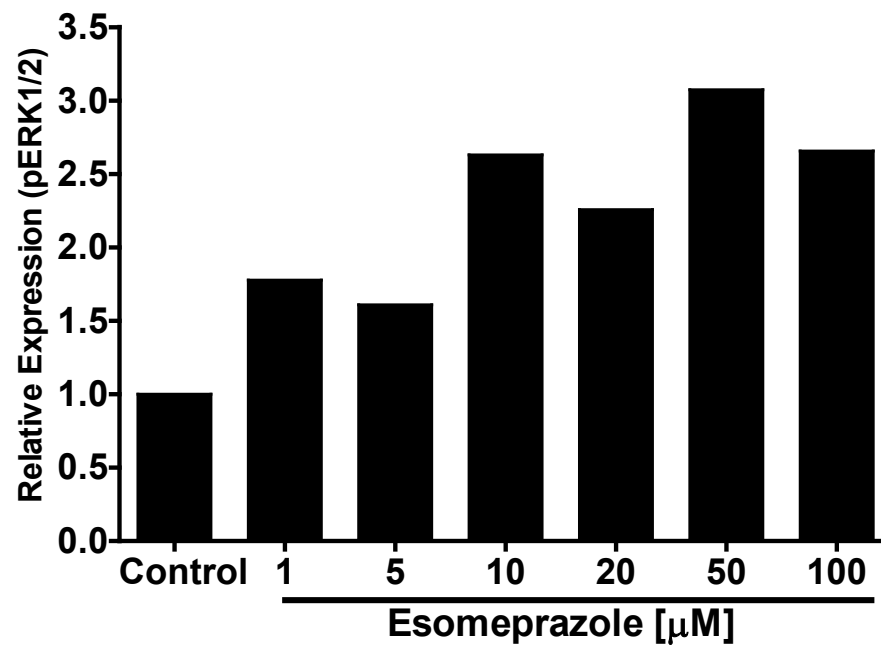

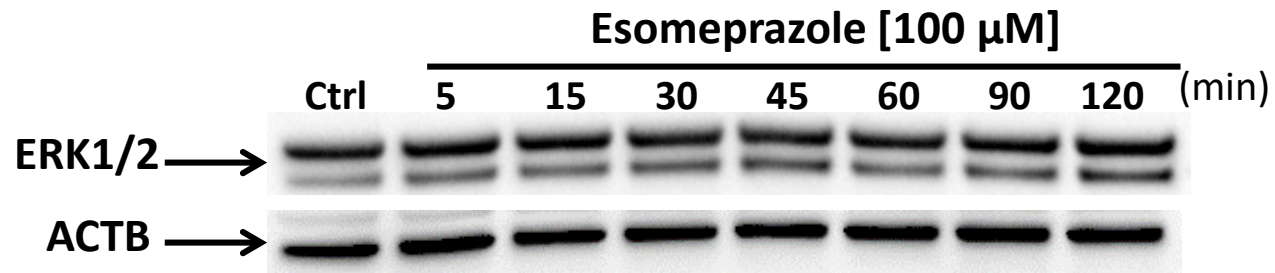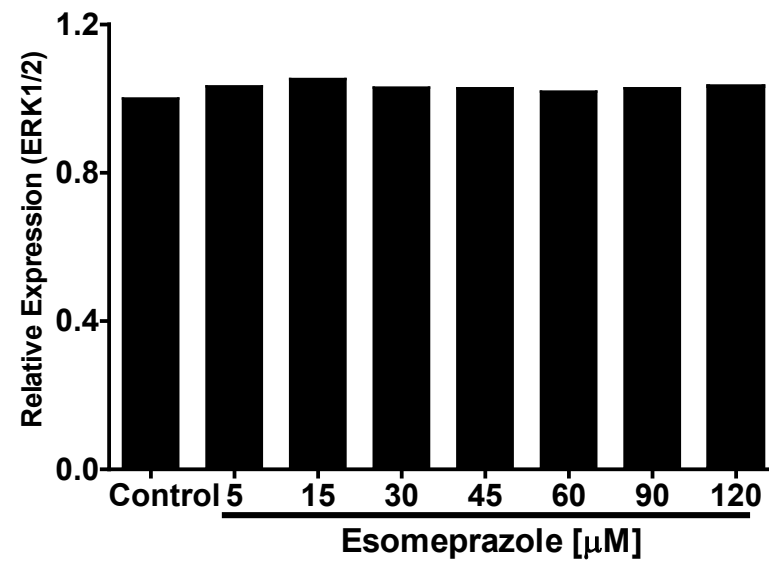

Figure S8

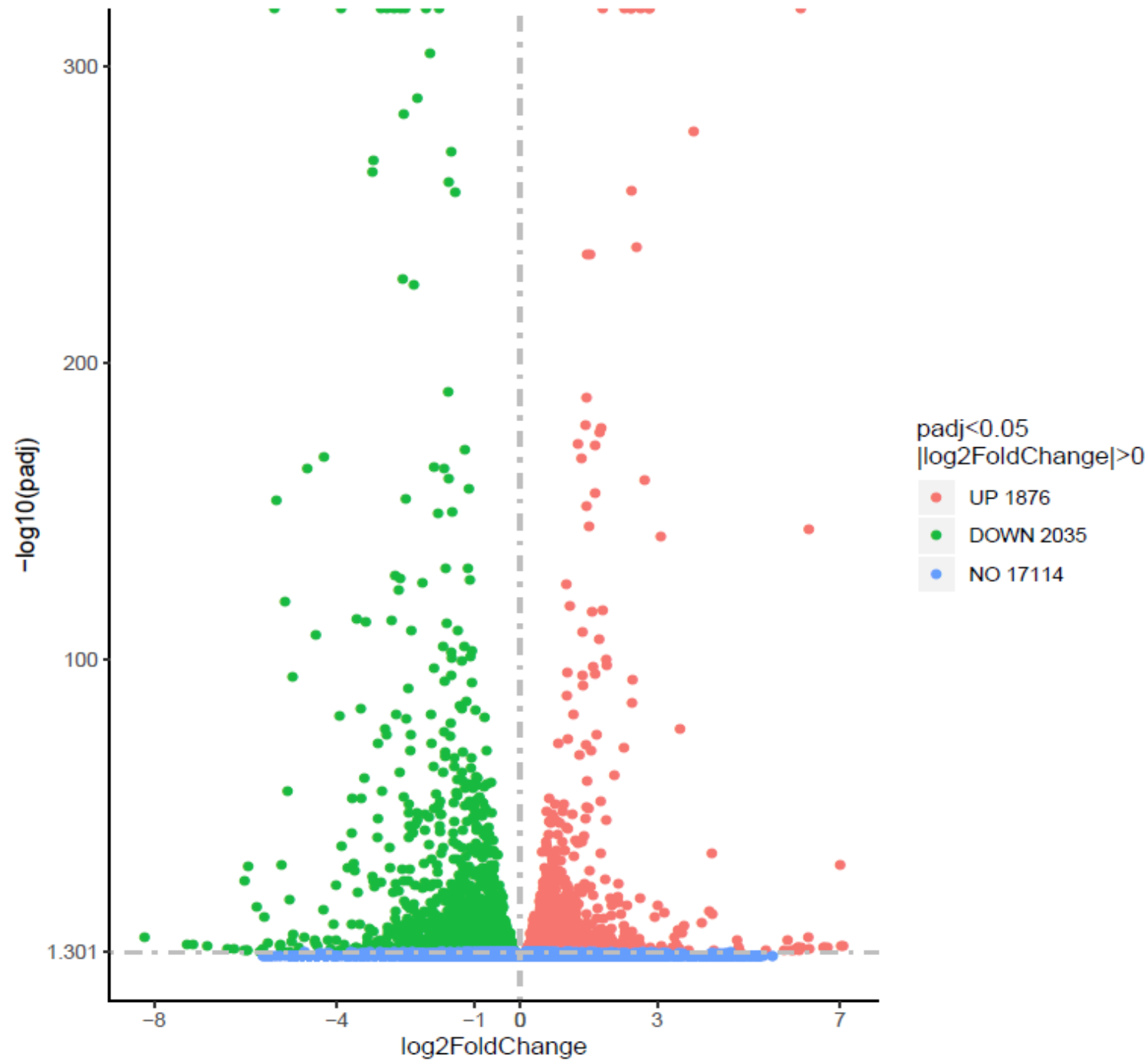

Supplement: Supplementary file 4 — Additional file 4: Figure S1. Immunofluorescence data showing increased expression of heme oxygenase 1 (HO1) protein by esomeprazole in primary human lung epithelial cells. The cells were treated with various concentrations of esomeprazole for 24 hours (1-100 μM) prior to staining with mouse anti-HO1 antibody (shown in red). The cell membrane was stained with Alexa Fluor 488-conjugated phalloidin and is shown in green. Figure S2. Western blot data showing nuclear translocation of nuclear factor-like 2 (Nrf2) in human lung endothelial cells. The cells were treated for 24 hours with esomeprazole (1-100 μM) or vehicle control (water) prior to isolation of nuclear protein. Data is representative of five independent experiments. Histone H3 is used as a loading control. Densitometric quantification of the protein bands relative to the housekeeping control protein histone H3 is shown in the lower panel. Figure S3. Quantitative RT-PCR (qRT-PCR) data showing dose-dependent upregulation of NADPH quinone oxidoreductase 1 (NQO1) by esomeprazole in human IPF lung fibroblasts. The cells were treated with various concentrations of esomeprazole for 6 hours prior to isolation of RNA for qRT-PCR. Data is Mean ± SEM from triplicate experiments. *p<0.05 compared to control. Figure S4. Western blot data showing no change in the protein expression of Kelch ECH associating protein 1 (Keap1) by esomeprazole in human lung epithelial cells. The cells were treated for 24 hours with various concentrations of esomeprazole (1-100 μM) or vehicle control prior to isolation of total protein. Data is representative of three independent experiments. Beta actin (ACTB) is used as a loading control. Densitometric quantification of the protein bands relative to ACTB is shown in the lower panel. Figure S5. Western blot data showing phosphorylation of ERK1 and ERK2 in human lung epithelial cells treated with vehicle control or various concentrations of esomeprazole (1-100 μM) for 24 hours. Data is represen [file 12950_2021_284_MOESM4_ESM.pdf]
